# Probiotics for the Control of Helminth Zoonosis

**DOI:** 10.1155/2018/4178986

**Published:** 2018-01-31

**Authors:** Abadi Amare Reda

**Affiliations:** School of Veterinary Medicine, Wollo University, P.O. Box 1145, Dessie, Ethiopia

## Abstract

This paper is a comprehensive, concise, and an up to date review about probiotics effect and mechanisms against helminth infections of zoonotic importance. Zoonoses are diseases that can be transmitted from animals to humans in a reversible way. Despite zoonotic helminth diseases being still a challenge to the public health and the agriculture industries globally, they were still neglected in both human and veterinary medicine. Moreover, the increasing emergence of anthelmintic drug resistance constitutes failures of most disease control strategies, alarming for a quest to new alternative control approaches. Consequently, the use of beneficial microorganisms, probiotics, is becoming interesting for its prophylactic or therapeutic application against several diseases including helminths. Recent studies on probiotics against parasites and the interactions between bacteria, parasites, and the immune system in the gut draw much attention. However, the effects of these beneficial microorganisms in helminth infections remain largely unexplored. Therefore, the aim of the present review is to raise attention and to summarize recent findings on probiotics research against helminth parasites of zoonotic significance. State-of-the-art research on beneficial effects of bacteria on helminth infections and their proposed mechanisms of action is thoroughly discussed.

## 1. Introduction

Zoonosis is an infectious disease that can naturally be transmitted through direct or indirect means from animals to humans, or vice versa. These infections can be caused by bacteria, viruses, fungi, parasites, and prions. People may acquire these harmful agents from infected animals by several ways. For instance, infection can be via direct contact with feces, handling of pets, ticks, or mosquito bites, or via consumption of undercooked food of animal origin. Currently, more than 200 pathogens are being regarded as zoonoses. Possible driving factors for the emergence of zoonoses are global travel, international trades, and climate change, among others. As a result, the magnitude of these diseases may augment as long as these driving factors continue to amplify. Consequently, zoonotic diseases remain a global public health threat today [1, 2].

Nowadays, one of the most prevalent zoonotic diseases is infection with helminth parasites, which infect about one-third of the human population worldwide. Helminths are parasitic worms, an evolutionarily ancient and diverse group of metazoan organisms, which include cestode tapeworms, nematode roundworms, and trematode flukes. Infection with helminths usually tends to be chronic rather than acute infection, although there can be acute manifestations after initial infection in naive hosts. Mortality is low in healthy hosts, but is often life-threatening to individuals with poor immunity. However, morbidity can be quite high. Mental and growth stunting among children is also a big problem with helminth infections. Hence, helminth parasites are of significant concern to public health and food safety. Furthermore, helminths also infect a wide range of animal species and bring about direct and indirect economic losses to livestock production [3]. Prevention and control of helminth parasitic zoonosis is possible, from a simple application of hygiene and sanitation to regular deworming with anthelmintic drugs. However, due to the absence of effective vaccines and the emergence of anthelmintic drug resistance, eradication of parasitic infestation still lingers a challenge, which requires the development of new alternative strategies. Thus, the interest in exploiting probiotics as an alternative to drugs has increased considerably during the last couple of years.

Probiotics are exogenous living microorganisms, which are beneficial to the host's health when administered in the digestive tract. The most widely used microorganisms for this purpose are bacteria of the genus* Lactobacillus* and* Enterococcus*, and some fungi and yeasts [4]. The protective effect of probiotics is by competitive exclusion or colonization resistance of pathogenic microorganisms in the gut. Another mechanism is their ability to produce antibacterial substances, like bacteriocins or oxygen peroxide, or by immunomodulation [5]. Likewise, probiotics may interfere with the physiology of parasites in the gut. Furthermore, their secretions may have anthelmintic effects and can reduce the virulence of many parasites. Hence, probiotics can be an integral part of helminth parasite control strategies [6].

Recent studies on probiotics against parasites and the interactions between bacteria, parasites, and the immune system in the gut draw much attention [7–10]. However, effects of probiotics on helminth infections remain largely unexplored. Thus, the aim of the present review is to compile recent research findings on probiotics against helminth parasites of zoonotic importance. In this review, state-of-the-art research on beneficial effects of bacteria on helminth infections and their proposed mechanisms of action will be thoroughly discussed.

## 2. Trends of Probiotics against Helminth Zoonosis

Zoonotic helminth infections are still remaining a challenge posing a significant impact on public health, food safety, and agriculture industries worldwide [11]. Despite many anthelmintic drugs being commercially available, resistance rates are increasing, alarming for a search for new alternate therapeutic strategies. As a result, the use of beneficial microorganisms, probiotics, is becoming interesting for its prophylactic or therapeutic application against several diseases including helminths. Recent studies on probiotics against parasites and the interactions between bacteria, parasites, and the immune system in the gut showed promising results. However, the effects and mechanism of these beneficial microorganisms in helminth infections remain incompletely understood. Therefore, it is imperative to recognize the current trends in probiotic research done on helminths thus far to better explore the mode of action and its beneficial effect on helminths. This review was developed based on state-of-the art of beneficial bacteria research on helminths, mainly schistosomiasis, trichinellosis, toxocariasis, trichuriasis, ascariasis, hookworms, and* Strongyloides*, and discussed accordingly.

### 2.1. Probiotics against Schistosomiasis

**Figure 1:**
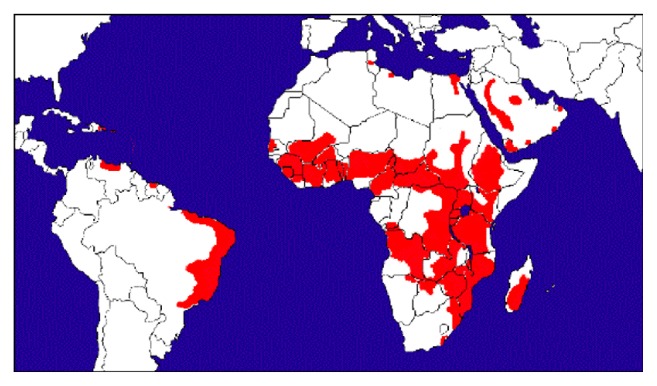
Global distribution of schistosomiasis (areas in red color) due to* S. mansoni*. Source: http://www.infectionlandscapes.org/2012/06/schistosomiasis.html.

Zoonotic schistosomiasis is caused by trematodes of the genus* Schistosoma*, mainly by* S. mansoni*,* S. japonicum,* and* S. mekongi* [11]. Other less prevalent species like* S. haematobium*,* S. guineensis*, and* S. intercalatum* can cause systemic diseases in people. However, most zoonotic cases of schistosomiasis are attributed to* S. japonicum* [12]. The parasite is widely distributed throughout tropical and subtropical areas. It is the third most devastating neglected tropical disease in the world with an overall disease burden of 3.31 million disability-adjusted life year (DALY) [3]. Despite only 14% of global schistosomiasis being of zoonotic origin, the global burden of zoonotic schistosomiasis is estimated to be over 10 million DALYs per annum [11]. More than 258 million people are infected in 78 endemic countries worldwide, of which 92% of them live in Africa [13]. A map showing the global distribution of human schistosomiasis due to* S. mansoni*, which were developed by the Schistosomiasis Research Group at Cambridge University, is depicted in Figure 1.

Pathogenesis of human schistosomiasis begins after the larval stage of the parasite is transmitted via skin penetration when people are doing their routine activities in infested water areas. Thereafter, the larvae grow into adult stage and reside in the blood circulation, where female worms release eggs. The eggs that are not excreted spread and remain attached in body tissues thereby resulting in an immune system reaction and gradual damage to organs. Mental and growth stunting among children is a big problem with infections by this helminth. Also adults are as likely to become infected and can show a reduced ability to work. In chronic cases, the parasite can also damage the liver, intestine, spleen, lungs, and bladder [14]. Mass drug administration of praziquantel has been the main means of control so far, but there are complaints with this approach such as drug resistance. Furthermore, vaccines are in various stages of development today [15, 16]. Thus, considering the multifaceted socioeconomic impact of zoonotic schistosomiasis, the search for safe and more effective control remedies is required.

To date, various attempts have been made to investigate the protective and curative effects of beneficial bacteria in mice models for use in the control of* S. mansoni* [17–21]. Several probiotic strains, like* Zymomonas mobilis*, probiotic labneh containing* Streptococcus salivarius *subsp*. thermophilus*,* Lactobacillus delbrueckii *subsp*. bulgaricus,* and different* Lactobacillus* species, have been evaluated. Their anthelmintic and immunomodulatory effects on* S. mansoni* are summarized in Table 1. For instance,* Lactobacillus sporogenes* is among the most commonly studied [20, 21] probiotic strains that showed a significant antischistosome effect in egg and larval stages of the parasite. It has remarkably reduced the worm burden as well as egg count. Interestingly, both authors have reported that* L. sporogenes* reduced chromosomal aberrations and DNA damage induced by infection in the host.

### 2.2. Trichinellosis

Trichinellosis is among the top 10 global ranking of food borne parasitic infections, which pose a public health threat and economic losses in pig production and food safety worldwide [22]. Globally, trichinellosis has been reported in over 55 countries, and an estimated 10,000 cases occur every year with 0.2% of these cases being lethal [23]. Humans can be infected by many species of* Trichinella* including* T. spiralis*,* T. britovi*,* T. murrelli*, and* T. nativa* [24]. However, the most important etiological agent to cause disease in people worldwide is* T. spiralis*, the species most commonly found in pigs [25]. Other* Trichinella* species are less commonly reported and may be found in some parts of the world, usually infecting wild animals.

Ingestion of uncooked infected meat from pigs is the main source of infection in humans. Occasionally, horses and other domestic animals infected with larvae of* Trichinella* may also infect people [25]. The disease in humans is characterized by enteritis (intestinal phase) and tissue inflammation in the skeletal muscles with degenerative changes (tissue/muscular phase). The pathogenesis of* T. spiralis* infection is mainly attributed to the formation of larval capsules and host immunosuppression [26]. The latter could be regulated by a serine protease from adults and newborn larvae in the intestinal and in the muscular phases [27]. Moreover, the parasite can alter dendritic cell function and induce immunosuppression by regulatory T and B cells, stimulated macrophages, and cytokine production [28]. Nevertheless, the molecular mechanisms mediating these processes remain unknown.

Treatment of human trichinellosis with anthelmintics is not effective against all developmental stages of the parasite as it is only effective for adult worms. Furthermore, endeavors made thus far to produce vaccines against trichinellosis have not been successful due to the wide range of species-specific antigens and immunosuppressive effects of host responses [29]. Alternatively, the use of the immune stimulating probiotic bacteria has been suggested [7, 30].

In several studies* T. spiralis* has been used as a model parasite to validate anthelmintic and immunomodulatory properties of probiotic and bacteriocin-producing bacterial strains [7, 8, 30–32]. In all studies, the most widely explored bacteria are from the genus* Lactobacillus*, of which,* Lactobacillus casei* is the top ranked strain. It has anthelmintic effect with an efficacy range from 75% to 100% protection. Another bacterial strain within the genus* Lactobacillus*, which has showed a remarkable degree of protection around 90% against* T. spiralis*, is* Lactobacillus plantarum P164* [7]. This suggested that these aforementioned Lactobacillus strains may be safe to use as prophylactic or curative probiotics against* T. spiralis*. Besides their anthelmintic effect, most of the aforementioned probiotic strains influence the innate immune system such as phagocytosis (Table 2).

Other probiotic strains stimulate the production of IgG and IgA anti-*T. spiralis*, which help maintain intestinal humoral immunity by attaching to antigens, thus preventing attachment to the epithelium. Moreover, a more recent development by Dvorožňáková et al. [8] reported that the highest stimulatory effect on phagocytic activities of blood monocytes and leukocytes and their enzymatic activity was induced by strains* Enterococcus durans ED26E/7*,* L. fermentum AD1*, and* L. plantarum 17L/1*. This may suggest how these probiotic strains act and the interactions between the parasites and the bacteria by stimulating the immune cells and their enzymatic activity.

### 2.3. Toxocariasis

Toxocariasis is a neglected roundworm parasitic zoonotic infection distributed among many countries throughout the world [33]. It can be caused by* Toxocara canis* and* Toxocara cati*, which are the natural inhabitants of the intestines of dogs and cats, respectively. The most common Toxocara parasite of concern to humans is* T. canis*. It is associated with visceral larva migrans, which is characterized by the migration and permanence of larvae of helminths in humans [34]. The epidemiology of toxocariasis is worldwide, and prevalence rates can reach as high as 40% or more in parts of the world [35]. Humans can be infected either by accidentally ingesting infected eggs or eating undercooked or raw meat from an infected paratenic host like chickens, ruminants, or pigs [36, 37]. Once inside the body, the eggs hatch in the small intestine and the larvae penetrate the wall and spillover to different organs and tissues via the blood circulation [38].

Even though toxocariasis in most human cases is asymptomatic, the migrated larvae can end up in the liver, lungs, heart, and brain causing severe complications. The two most common classical forms of the disease in people are visceral larva migrans (VLM) and ocular larva migrans (OLM) [39]. Besides, other forms like covert toxocariasis (CT) and neurological and asthmatic forms of toxocariasis have been documented [40]. However, the mechanism of how these roundworms invade the host and modulate their immune system is unknown. Thus, further studies on the interactions of this parasite with the immune system and gut flora in the host are needed to advance the knowledge about immune protection against* T. canis* [41]. The prevention and control of toxocariasis in the definitive host, that is, dogs and cats, will reduce the risk of infection for humans and other paratenic hosts. However, treatment is difficult due to the occurrence of different clinical forms of human toxocariasis [42]. Currently, new alternatives, like probiotics, are promising to control this zoonotic parasite.

Many studies have been attempted to evaluate the protective effects of probiotics against* T. canis* in mice experiments. Basualdo et al. [43] reported a significant reduction (75–100%) of worm burden in mice treated with a dose of 3 × 10^8^ (CFU/ml) of* Enterococcus faecalis*. Moreover,* E. faecalis CECT71219* at different doses of 7 × 10^4^ (CFU/g), 1.46 × 10^4^ CFU in culture and 1 × 10^8^ CFU fed in mice showed both in vitro and in vivo larvicidal activity [44]. In contrast, Avila et al. [45] reported that none of the* Saccharomyces boulardii* and* Bacillus cereus *var*. toyoi* showed in vitro effects against* T. canis* larvae. Interestingly, a recent study by de Avila et al. [46] has declared a definitive efficacy of supplementation with the probiotic* S. boulardii* at a dose of 1 × 10^7^ (CFU/g), which reduced the intensity of infection in mouse studies. Besides the anthelminthic effect,* S. boulardii* modulates the mRNA expression levels of especially interleukin- (IL-) 12 and interferon gamma (IFN-*γ*) in mice. However, to understand the molecular mechanisms of probiotics in this nematode infection further study is needed.

### 2.4. Trichuriasis

After ascariasis and hookworm infections, trichuriasis also called whipworm infestation is the world's third widespread nematode affecting around 800 million people and a range of mammalian hosts [47]. It remains a public health risk as it causes a huge economic burden and decreases the quality of life for many people in developing countries [48]. The causative agents of zoonotic trichuriasis are* Trichuris vulpis* and* T. suis*, which are whipworms of dogs and pigs, respectively. Whereas* T. trichiura* is a species that parasitizes humans, it can also be found in chimpanzees, monkeys, and lemurs. Despite its evolutionary relationship with* T. suis* found in pigs, there is no evidence that its transmission is zoonotic, except in unusual circumstances [49]. Most recent studies [50, 51] found no genetic difference between* T. trichiura* and* T. suis* from* Trichuris* samples collected in humans and pigs in Africa, Asia, Europe, and the New World and suggesting a common African origin of the parasite.

Dogs and other wild canids and, possibly, pigs are the major reservoirs of zoonotic species of* Trichuris*. The parasites spread from person to person via the ingestion of eggs via food or water, or via hands contaminated with infective eggs [49]. Most cases of human infection with zoonotic* Trichuris* have been asymptomatic or may show moderate diarrhea. Ingestion of* T. suis* eggs results in short term self-limited colonization of humans [52]. Regular deworming with anthelminthic drugs such as albendazole and mebendazole and high-standard hygienic measures may lessen infections. Nevertheless,* Trichuris* could persist in the animal host and soil due to their egg being highly resistant and long lifespan of adult worms. Moreover, mass drug administration (MDA) of suboptimal drug dosage is the perfect “breeding ground” for drug resistance. Thus, eradication of trichuriasis requires a specific treatment strategy such as immune stimulant probiotics.

Several studies in mice have revealed the effects of beneficial bacteria and associated interactions in a model of enteric nematode infection with the intestinal whipworm* T. muris* [53–55]. Oral supplementation with live* Lactobacillus rhamnosus (JB-1)* at a dose of 1  × 10^9^ CFU/day has significantly accelerated larvae removal in* T. muris* resistant C57BL/6 mice. This was accompanied by upregulation of anti-inflammatory cytokine IL-10 levels and mucus secreting epithelial cell numbers. These findings revealed that probiotics such as* L. rhamnosus (JB-1)* modulate the number of mucus secreting epithelial cells and enhance worm removal through an interleukin (IL-10)–goblet cells-mediated pathway [54].

In contrast, a report by Dea-Ayuela et al. [53] showed that oral consumption of* L. casei* ATCC7469 increased susceptibility to infection with* T. muris*. This finding was associated with down-regulation of Th1 immune response with low levels of gamma interferon (IFN-*γ*) and Th2 response characterized by decline levels of IL-4 and IL-13 [53]. Furthermore, Holm et al. [55] reported that persistent* T. muris* infection remarkably enhances the population of the genus* Lactobacillus*, but causes a reduction in the population of other bacterial species in the gut. Thus, the effects of interactions between* T. muris* and the microbiome in the host can be aimed at promoting mutual benefit, or elimination of one another [56, 57]. Studies showing helminth infection increasing gut diversity would be interesting if helminths can in fact be commensal and promote growth of “good” gut bacteria. Currently, there have been a few trials with human infections of* Trichuris* to treat various inflammatory bowel diseases (IBD). Nowadays, experimental and clinical trials with* T. suis* both in vitro and in vivo showed various immune regulatory strategies and promoted host immune responses. This property of the parasite may help to counteract many diseases like Crohn's disease [52] and multiple sclerosis [58, 59].

### 2.5. Ascariasis

Ascariasis is the most common soil-transmitted roundworm zoonotic infection.* A. lumbricoides* and* A. suum* are phylogenetically related species that infest people and swine, respectively [60].* A. lumbricoides* has a prevalence rate of 25% and usually affects humans worldwide, but most frequently occurs in tropical and subtropical areas [61, 62]. Whereas* A. suum* commonly infects pigs globally and causes huge economic losses to the pig industry. Humans can be infected by ingestion of infective* A. suum* eggs present in soil especially where pig manure is widely used as fertilizer [63–67]. Most recently, incidence rates of 13.2% of* A. suum*-specific antibodies in humans were reported [67]. Taking into account its global distribution and huge impact on public health and economy, appropriate invasive control strategies are required to control ascariasis.

Regarding probiotics on* A. suum*,* Bifidobacterium lactis subspecies animalis* [68] and* Lactobacillus rhamnosus* [69, 70] have been reported so far. Both bacterial strains have reduced* Ascaris suum*-induced eosinophil activity and decreased the severity of allergic skin and lung responses in pig models (Table 3). Thus, these study protocols could be used to validate the effect of different probiotic strains on responses to different pathogens to reduce drug resistance of Ascaris species.

### 2.6. Other Helminths

In addition to the aforementioned helminth infections, other roundworms, like hookworms and* Strongyloides*, are more prevalent helminth zoonotic infections causing huge morbidity and economic burdens worldwide. Globally, around 576–740 million and 30–100 million people are infected by hookworms and* Strongyloides*, respectively [71]. Among hookworms,* Ancylostoma braziliense* is regarded as the most common cause of cutaneous larva migrans in humans. Other species including* A. caninum*,* A. ceylanicum*,* Uncinaria stenocephala*, and* Bunostomum phlebotomum* are involved less frequently. Moreover,* A. ceylanicum* is the only zoonotic hookworm known to produce patent intestinal infections in humans. More recently, a number of studies have been reported looking at molecular diagnosis of zoonotic* A. ceylanicum* in humans and dogs in different parts of the world [72–77]. Despite* A. caninum* being the most widely distributed among hookworms, it infrequently causes eosinophilic enteritis in humans [78]. Regular deworming of dogs and cats with a range of antinematode drugs can reduce the risk of infection in humans [71]. Nevertheless, resistance has been observed in some of the currently used drugs such as pyrantel in dogs [78]. Hence, novel control approaches such as probiotics may confer sustainable protection against hookworms.

A “pool” of 1 × 10^6^ CFU of each strain of* L. acidophilus*,* L. plantarum*, and* L. delbrueckii* have shown a significant effect on* A. caninum* infection with around 90% efficacy in naturally infected dogs. Moreover, an increase in leukocyte and lymphocyte counts was reported [79], suggesting the immune activation effects of probiotics. On the other hand,* Bifidobacterium animalis strain 04450B* at dose of 2 × 10^9^ CFU revealed a much lower response with 33% reduction of adult worms and 21% reduction of egg production in* Strongyloides venezuelensis* infected mice [80].

## 3. Mechanisms of Action of Probiotics

The efficacy of beneficial bacteria on the host often depends on the mechanism by which they exert their activity. They may involve one or multiple modes of action including production of antimicrobial substances, modulation of the mucosal immune system, alteration of the intestinal microflora, and enhancement of enzymatic activity [81]. The primary mode of action of probiotics against parasites might be by enhancing the intestinal barrier and modulation of the microflora in the gut [8, 9, 44–46, 55]. They may augment the number of beneficial microorganisms, like lacto-bacilli and bifidobacteria, which then inhibit growth of harmful pathogens by competing for attachment site in the intestinal mucosa. The second proposed mechanism may involve secretion of antimicrobial substances, like bacteriocins, and organic acids such as lactic, acetic, and butyric acid, mainly secreted by* Lactobacillus* species and may have a larvicidal effect on parasites [82].

Immunostimulation and immunomodulation of either innate or adaptive immune system components [7, 8, 30, 46] are among the leading proposed elucidations for how probiotics exert their action against helminths. For example, probiotic* S. boulardii* promoted a reduction in intensity of infection by* T. canis* by modulating cytokine mRNA expression, especially IL-12, in experimentally infected mice [46]. Furthermore,* L. sporogenes* act against cytokine induced apoptosis by decreased chromosomal aberrations and DNA damage in* S. mansoni* infected mice [20, 21]. Nevertheless, modes of action of specific probiotics are generally not understood. Interestingly, effects of probiotics are the product of cross-talk between host and probiotic agent. Thus, more research on host-microbes or pathogen-pathogen interactions using state-of-the-art immunogenetic technologies may perhaps illuminate our knowledge of probiotics mode of action on helminths [81].

## 4. Conclusions

Considering the multifaceted socioeconomic consequences of zoonotic helminth infections and increasing rates of anthelmintic drug resistance, a quest to new alternative control strategies, like probiotics, is urgently needed to mitigate infection. The efficacy of probiotics strains, mainly bacteria in the genera* Lactobacillus*,* Enterococcus,* and* Bifidobacterium*, has been largely evaluated mainly for the control of schistosomiasis, trichinellosis, and toxocariasis. A difference in the efficacy of these strains, which might be attributed to the variability in study design, experimental animals used, dose ranges, and route of administration, was discerned. Results from these experiments indicated that some bacterial strains in the genera* Lactobacillus* and* Enterococcus* could be used as prophylactic or curative probiotics against helminths after validating it in repeated human and animal clinical trials. Their mode of action can be strain-specific or by a combination of different mechanisms. Furthermore, most effects of probiotics on helminths have been conducted in animal experiments and in vitro culture. Studies involving human trials were scarcely reported. In some cases, helminth-microbe interactions were also assessed. Nevertheless, the molecular mechanisms whereby these beneficial microorganisms act remain poorly understood. Hence, further investigations on host-microbe or pathogen-pathogen interactions using modern molecular techniques could enlighten our knowledge of the mechanism of action of probiotics.

## Figures and Tables

**Table 1:** Probiotic strains used against *Schistosoma mansoni* infection in mice.

Probiotics strain	Dose/route	Mechanisms	Antiparasitic effect	References
*Zymomonas mobilis*	1 × 10^9^ CFU/mL orally, at a dose of 0.3 mL/day	Provoke a secondary immune response	A 61% protection from the infection was observed in the treated group	[17]

Probiotic labneh containing *streptococcus salivarius subsp. thermophilus*, *Lactobacillus delbrueckii subsp. Bulgaricus* and DVS-ABT2	Probiotic labneh and garlic and onions fed for 21 days before and 45 days after infection	Improving intestinal balance	50%–66% reduction in worm burden; 70% and 56.44% egg count reduction in liver and intestine, respectively	[18]

*Lactobacillus casei B-444*; *Lactobacillus plantarum B-531*; *Lactobacillus reuteriB-14141* and *Lactobacillus acidophilus*	1 × 10^6^ CFU each mixed with feed	A significant stimulation for IgM response against SWAP before and after infection	Increased IgM; A decrease in the activity of AST, LDH and *γ*GT	[19]

*Lactobacillus sporogenes*	12.5 million spores/mice/week for 8 weeks orally	Decreased cytokine-induced chromosomal aberrations and DNA damage	Significant reduction in chromosomal aberrations	[20]

*Lactobacillus sporogenes*	12.5 million spores/mice/week for 8 weeks orally from the first day of infection	Reduced DNA damage; ameliorates the hepatic and intestinal damage	Reduced worm and egg count	[21]

SWAP: soluble worm antigen preparation, AST: aspartate transaminase, ALT: alanine transaminase, LDH: lactate dehydrogenase, *γ*GT: gamma-glutamyl transferase, DVS-ABT2: containing *Streptococcus thermophilus*, *Lactobacillus acidophilus,* and *Bifidobacterium bifidum*, and CFU: colony forming units.

**Table 2:** Effects of different strains of probiotics on *Trichinella spiralis* in mice model.

Probiotics strain	Dose/route	Mechanisms	Antiparasitic effect	References
*Lactobacillus acidophilus* P110, *Lactobacillus plantarum* P164 and *Lactobacillus casei* ATCC 7469	1.0 ml/kg/day with a concentration of 1.9 × 10^9^ CFU/ml orally	Both showed higher levels of IFN-*γ*	60.98%, 87.92% and 74.88% larval count reduction, respectively	[7]

*Enterococcus faecium *AL41, *Enterococcus durans* ED26E/7, *Lactobacillus fermentum *AD1 and *Lactobacillus plantarum* 17L/1	10^9^ CFU/ml in 100 *μ*l orally	Stimulated phagocytosis and respiratory burst of blood PMNL and high intensity of enzymatic stimulation	Protective effect was induced by all strains, the highest reduction by *E. faecium* AL41	[8]

*L. casei strain ATCC 469*	1.9 × 10^9^ CFU/ml orally	Reduced invasion of larvae into the host	Significant protective response	[31]

*Lactobacillus casei*	Intraperitoneal	Higher levels of IgG and IgA anti-*T. spiralis* and IL-4, but lower levels of IFN-*γ*	78.6%–100% protection	[30]

*Lactobacillus casei Shirota strain (LcS)*	Intraperitoneal	IgA anti-*T. spiralis* levels were higher	Induces protection against *T. spiralis*	[32]

PMNL: polymorphonuclear leukocytes.

**Table 3:** Probiotic strains used against *Ascaris suum *in pig model.

Probiotics strain	Dose/route	Mechanisms	Antiparasite effect	References
*Lactobacillus rhamnosus* (LGG)	1 × 10^10^ CFU/day orally	Induced an increase in toll-like receptor- (TLR-) 9 and tumor necrosis factor- (TNF-) *α* gene expression in AM	Reduced *Ascaris suum*-induced eosinophil activity in TBLNs	[70]

*Lactobacillus rhamnosus* HN001 (HN001)	1 × 10^10^ CFU/day orally	Increased in IFN-*γ* expression and regulatory (IL-10) cytokine expression	Decreased the severity of allergic skin and lung responses	[69]

*Bifidobacterium lactis *subspecies* animalis* (Bb12)	3.5 × 10^10 ^CFU orally	Increased mRNA expression of genes, including IL-25, but did not affect intestinal permeability	Did not interfere with normal expulsion of L4 from the jejunum	[68]

AM: alveolar macrophages and TBLNs: tracheobronchial lymph nodes.
